# Defining filled and empty space: reassessing the filled space illusion for active touch and vision

**DOI:** 10.1007/s00221-016-4673-x

**Published:** 2016-05-27

**Authors:** Elizabeth S. Collier, Rebecca Lawson

**Affiliations:** Department of Experimental Psychology, University of Liverpool, Eleanor Rathbone Building, Bedford Street South, Liverpool, L69 7ZA UK

**Keywords:** Touch, Vision, Kinaesthetic, Cutaneous, Filled space illusion, Length

## Abstract

In the filled space illusion, an extent filled with gratings is estimated as longer than an equivalent extent that is apparently empty. However, researchers do not seem to have carefully considered the terms filled and empty when describing this illusion. Specifically, for active touch, smooth, solid surfaces have typically been used to represent empty space. Thus, it is not known whether comparing gratings to truly empty space (air) during active exploration by touch elicits the same illusionary effect. In Experiments 1 and 2, gratings were estimated as longer if they were compared to smooth, solid surfaces rather than being compared to truly empty space. Consistent with this, Experiment 3 showed that empty space was perceived as longer than solid surfaces when the two were compared directly. Together these results are consistent with the hypothesis that, for touch, the standard filled space illusion only occurs if gratings are compared to smooth, solid surfaces and that it may reverse if gratings are compared to empty space. Finally, Experiment 4 showed that gratings were estimated as longer than both solid and empty extents in vision, so the direction of the filled space illusion in vision was not affected by the nature of the comparator. These results are discussed in relation to the dual nature of active touch.

## Introduction

Haptic perception has a dual nature which provides us with both cutaneous and kinaesthetic information (Lederman and Klatzky 2009; Dupin et al. 2015). Cutaneous cues result from direct input to receptors in the skin. Moving the body through air provides some cutaneous information, particularly if the movement is at speed. However, direct contact with a tangible surface provides stronger and more salient cutaneous cues. Kinaesthetic cues broadly refer to inputs from the musculo-skeletal system and arise primarily from limb movement (Dijkerman and de Haan 2007; Lederman and Jones 2011; Proske and Gandevia 2009, 2012). Actively exploring a smooth, solid surface provides both cutaneous and kinaesthetic information, whereas moving the body through still air provides mainly kinaesthetic information.

It was long held that cutaneous cues are less informative about spatial features than kinaesthetic cues (Gibson 1962; Magee and Kennedy 1980). For example, Bergmann Tiest et al. (2011) suggested that although observers can perceive and discriminate lengths when only cutaneous cues are present, haptic length perception is primarily based on kinaesthetic information. However, this view has recently been challenged by Van Doorn and colleagues, who have shown that cutaneous cues alone can be used to estimate length (Van Doorn et al. 2012a, b, 2013). Indeed, they reported that participants were more sensitive to differences in length between stimuli when using cutaneous information alone than when using kinaesthetic information alone.

Consistent with the claim that cutaneous cues are an important source of spatial information, participants have been shown to overestimate the distance they need to move their hand to reach a visual target when cutaneous feedback is withdrawn. Ebied et al. (2003) used anaesthesia to block cutaneous information to participant’s hands. This adversely affected participant’s ability to use a pen to follow the movements of a target on a screen. In the absence of cutaneous information, they overestimated how far they needed to move to reach the target each time it moved. Grip force on the pen was not affected by the anaesthesia, so this result did not appear to be simply due to an impaired ability to use the experimental equipment. Instead, length information acquired through cutaneous inputs may not be processed in the same manner as information acquired through kinesthesis (Jastrow 1886).

**Fig. 1:**
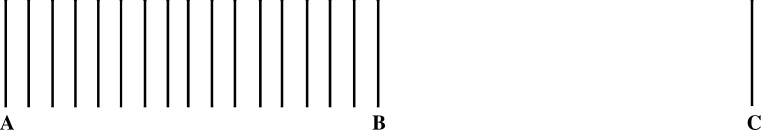
Typical configuration of the visual filled space illusion, where distance AB, filled with gratings, is perceived as longer than the unfilled, comparator distance BC. In fact, distances AB and BC are equal

The study of perceptual illusions allows us to better understand typical perception by analysing systematic biases that can occur (Coren and Girgus 1978). Illusions such as the filled space illusion (shown in Fig. 1) have been used to study biases in length perception in both vision (Coren and Girgus 1978; Eriksson 1970; Mikellidou and Thompson 2011, 2014) and touch (Dresslar 1893; Hayward 2008; Parrish 1893; Sanders and Kappers 2009; Suzuki and Arashida 1992).

Suzuki and Arashida (1992) directly compared the filled space illusion in vision and active touch. In both cases, extents filled with gratings were perceived as longer than smooth, solid surfaces and the strength of the illusion was comparable across the two modalities. Sanders and Kappers (2009) used the method of constant stimuli with a two-alternative forced-choice task to investigate the filled space illusion in active touch. On each trial, blindfolded participants compared the length of a smooth, solid surface to that of a solid surface filled with gratings with either a 4- or 8-mm spatial period. The two extents differed in length by 0.0, 0.8, 1.6, 2.4 or 3.2 cm. The filled space illusion was again obtained for touch: a smooth surface had to be physically longer to subjectively *feel* as long as gratings. The illusion was greater when the gratings were denser (1.1 cm for 4-mm gratings versus 0.9 cm for 8-mm gratings), matching the effect of changing the spatial period of the gratings on the visual filled space illusion (Coren and Girgus 1978).

The filled space illusion is typically described in terms of *empty* and *filled* space, where extent A–B in Fig. 1 is said to be filled (with gratings) and extent B–C is said to be empty. However, what defines an extent as “empty” is not always clear. In particular, for touch, researchers have often, without explanation, used a smooth, solid surface to represent empty space when they have investigated the filled space illusion. In both Sanders and Kappers’ (2009) and Suzuki and Arashida’s (1992) studies, the stimuli were made from swell paper where the gratings and solid surfaces were embossed to be raised by only a small amount from the surface of the paper. Participants therefore had continual access to both cutaneous and kinaesthetic information for all stimuli because their finger was always in contact with a solid surface. “Empty space” in these experiments might be better described as “saturated” space. Given the importance of cutaneous information to haptic spatial perception, as outlined above, this issue raises the question of whether the nature of the stimulus that gratings are compared to matters for the filled space illusion in touch.

To our knowledge, only Parrish (1893) has compared length estimations for gratings relative to truly empty space for which no cutaneous information is available. Parrish used wooden blocks with raised rubber bumps (ranging from two bumps, with one on each end of the block, up to nine bumps evenly distributed along the block). Blocks with two bumps represented empty space and blocks with nine bumps represented maximally filled space. The stimuli were pressed against the inside of the participant’s forearm, so they felt only the bumps and not the blocks. Participants kept their arm stationary and so kinaesthetic cues were not available. Parrish found the reverse of the standard filled space illusion: empty space was judged as longer than filled space. This reverse filled space illusion may signal that biases in length perception differ depending on the relative availability of cutaneous and kinaesthetic information. Parrish claimed that the filled space illusion reversed when kinaesthetic cues were not available. However, her participants had limited access to cutaneous as well as kinaesthetic cues so the reversal of the filled space illusion could have resulted from either of these differences, or both.

In summary, for the filled space illusion in touch (and also, as described later, in vision) experimenters do not seem to have carefully considered the nature of the comparator and a solid surface has often been used as the comparator to represent unfilled space. In touch, the distinction between a solid surface and truly empty space (air) may be particularly important because both cutaneous and kinaesthetic inputs provide length information and these inputs may be processed in different ways (Dupin et al. 2015; Ebied et al. 2003; Van Doorn et al. 2012a, b, 2013). In the present experiments, we investigated whether the nature of the comparator extent (whether a solid surface or empty space) that gratings are compared to influences the filled space illusion.

## Experiment 1

In this experiment, we tested whether the standard filled space illusion for active touch, namely that gratings feel longer than smooth, solid surfaces (Sanders and Kappers 2009; Suzuki and Arashida 1992), would reverse when people were asked to estimate the lengths of gratings compared to truly empty space. In contrast to Parrish (1893), but as in Sanders and Kappers (2009) and Suzuki and Arashida (1992), our participants actively explored stimuli in the same way on each trial and so the same kinaesthetic inputs were available for all stimuli. Cutaneous information was minimal when feeling the empty space extents but was present for the gratings and smooth, solid surface extents. We reasoned that if the filled space illusion in touch reverses as a result of a lack of movement, as Parrish (1893) proposed, then in this experiment the gratings should feel longer than both smooth, solid surfaces and empty space because people always actively explored the stimuli. In other words, we should find the standard filled space illusion irrespective of the nature of the comparator. If, however, the lack of cutaneous input caused the illusion to reverse in Parrish (1893) then we should find that participants overestimate the length of empty space compared to gratings even when they actively explore stimuli, just as Parrish found when her participants passively felt stimuli.

## Method

### Participants

Twelve participants (mean age = 20 years; 6 males) either volunteered or were given course credit for their time. Ethical approval for the present experiments was obtained from the relevant Research Ethics Committee at the Institute of Psychology, Health and Society at the University of Liverpool. All participants gave informed consent.

### Stimuli and apparatus

**Fig. 2:**
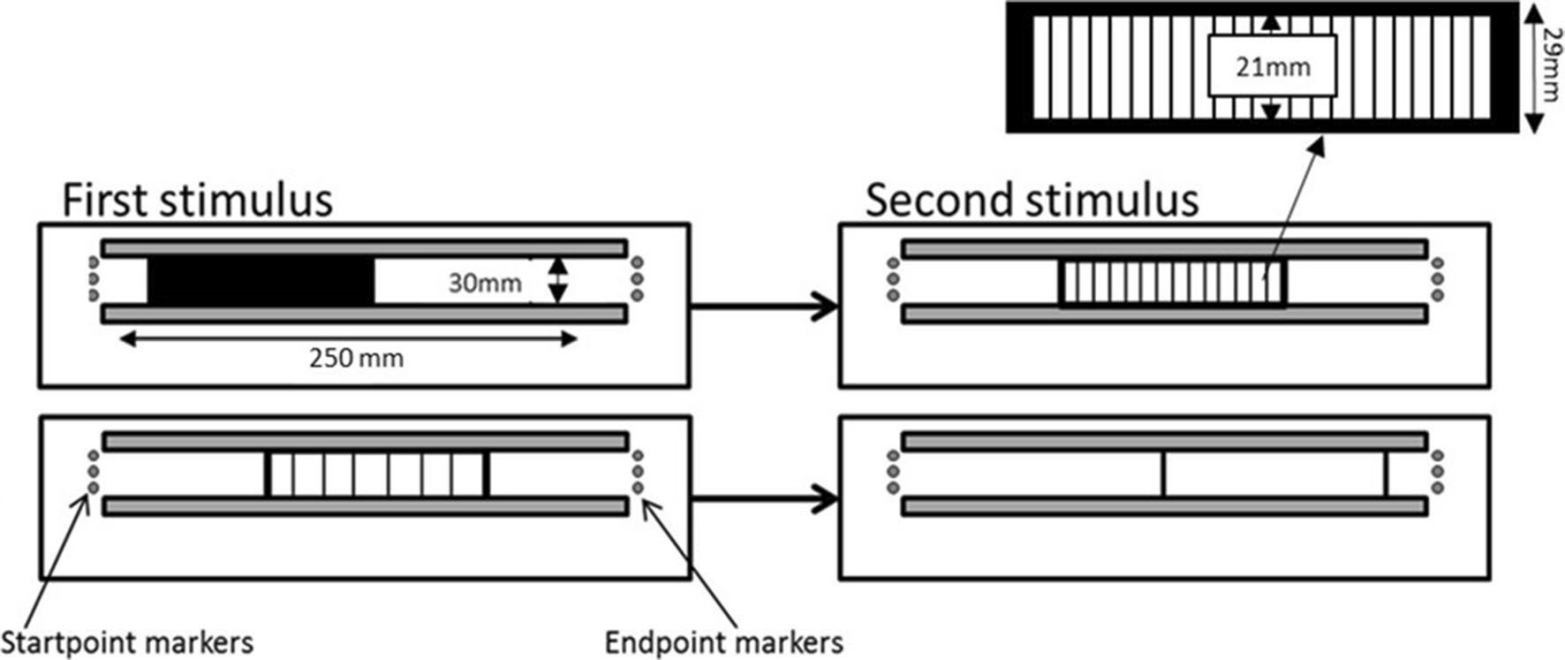
Setup for Experiments 1–3. The diagram shows two example trials using the four types of 12 cm stimuli: solid extents followed by 4-mm gratings (*top*) and 8-mm gratings followed by empty extents (*bottom*). The distance of each stimulus from the startpoint markers was varied by the experimenter so that the two successive stimuli on each trial were always placed in different positions

A laser cutter was used to create four sets of 0.5-cm-deep acrylic plastic stimuli: empty, solid, 4- and 8-mm gratings, as shown in Fig. 2. All stimuli were 29 mm tall and ranged from 9 to 15 cm in length in 1 cm increments. For the gratings, the extent between the start and end bars contained bars which were 1 mm wide and 21 mm high, with gaps of 4 or 8 mm between them. The 8-mm gratings were not used in Experiment 1. Sanders and Kappers (2009) noted that if the boundaries of unseen gratings explored by touch are not clearly distinguished from the grating bars, then participants may overestimate the length of gratings due to uncertainty about where they end. We therefore followed Sanders and Kappers and used a solid bar (width varying from 3 to 7 mm) to specify the start and end of the gratings and empty stimuli. Participants were told to consider the full length of the stimuli, from the start of the start bar to the end of the end bar when making their judgements.

Two 25-cm-long parallel tracks were fixed to a foam board surface on a table. There was a 30 mm gap between the tracks. On each trial, a stimulus was slotted between the tracks. The tracks ensured that the stimulus did not move during a trial. Three drawing pins at the left and right end of the tracks, 25 cm apart, marked the starting position of the participant’s index finger and its endpoint, respectively, as shown in Fig. 2.

### Design and procedure

Six participants were assigned to each of two groups. The 4-mm gratings were compared to empty stimuli for one group (the empty-gratings group) and to solid stimuli for the other group (the solid-gratings group). Participants were first shown the apparatus and examples of the stimuli (the 12-cm grating and the appropriate 12 cm comparator), and the task was explained to them. They were then blindfolded and were given four practice trials (comparing the 12-cm grating to the appropriate 12 cm comparator) before the experimental trials began. No feedback was provided during the practice trials.

On every trial, the first stimulus was inserted between the tracks and then the experimenter guided the index finger of the participant’s right hand to the startpoint. Participants then moved their index finger to the right until they reached the endpoint beyond the stimulus and then back again to the startpoint. The first stimulus was then replaced by the second stimulus and the procedure was repeated. Participants were asked to try and move their finger at a constant rate (though note that Sanders and Kappers (2009) found that average finger movement speed did not influence length estimates). They were not permitted to touch the base of the apparatus with their finger during exploration, and they were instructed to keep their hand raised to avoid doing so. Participants kept their elbow bent and were only permitted to rest their wrist on the edge of the tracks between trials. This meant that for empty stimuli, participants only received cutaneous input to their finger from the startpoint and endpoint markers and from the two bars marking the start and end of the stimuli. Participants responded by verbally stating which of the two stimuli felt longest, the first or the second. They received no feedback about their performance. To avoid participants using the distance between the stimuli and the start/endpoint markers as a cue for the length of the stimuli, the two stimuli to be compared on a given trial were always placed at different positions along the tracks, as shown in Fig. 2.

On every trial, a grating was compared to an empty stimulus for the empty-gratings group or to a solid stimulus for the solid-gratings group. The grating was presented first on half of the trials and second on the remaining trials, and on half the trials the grating was the 12 cm reference length and on the other half of trials the empty or solid stimulus was the 12 cm reference length. The available length differences were −3, −2, −1, 0, 1, 2 and 3 cm, which were defined as [empty/solid-gratings]. For example, for the solid-gratings group there were four trial types that all tested the length difference of −2 cm: these were solid 12 cm then grating 14 cm, solid 10 cm then grating 12 cm, grating 14 cm then solid 12 cm and, finally, grating 12 cm then solid 10 cm. Note that length difference was independent of whether the grating was presented first or second on a trial.

In total, participants completed 84 trials which tested each of the seven length differences three times in each of the four length difference trial types. The experiment lasted approximately 1 h and participants were offered a short break after 42 trials.

### Results and discussion

**Fig. 3:**
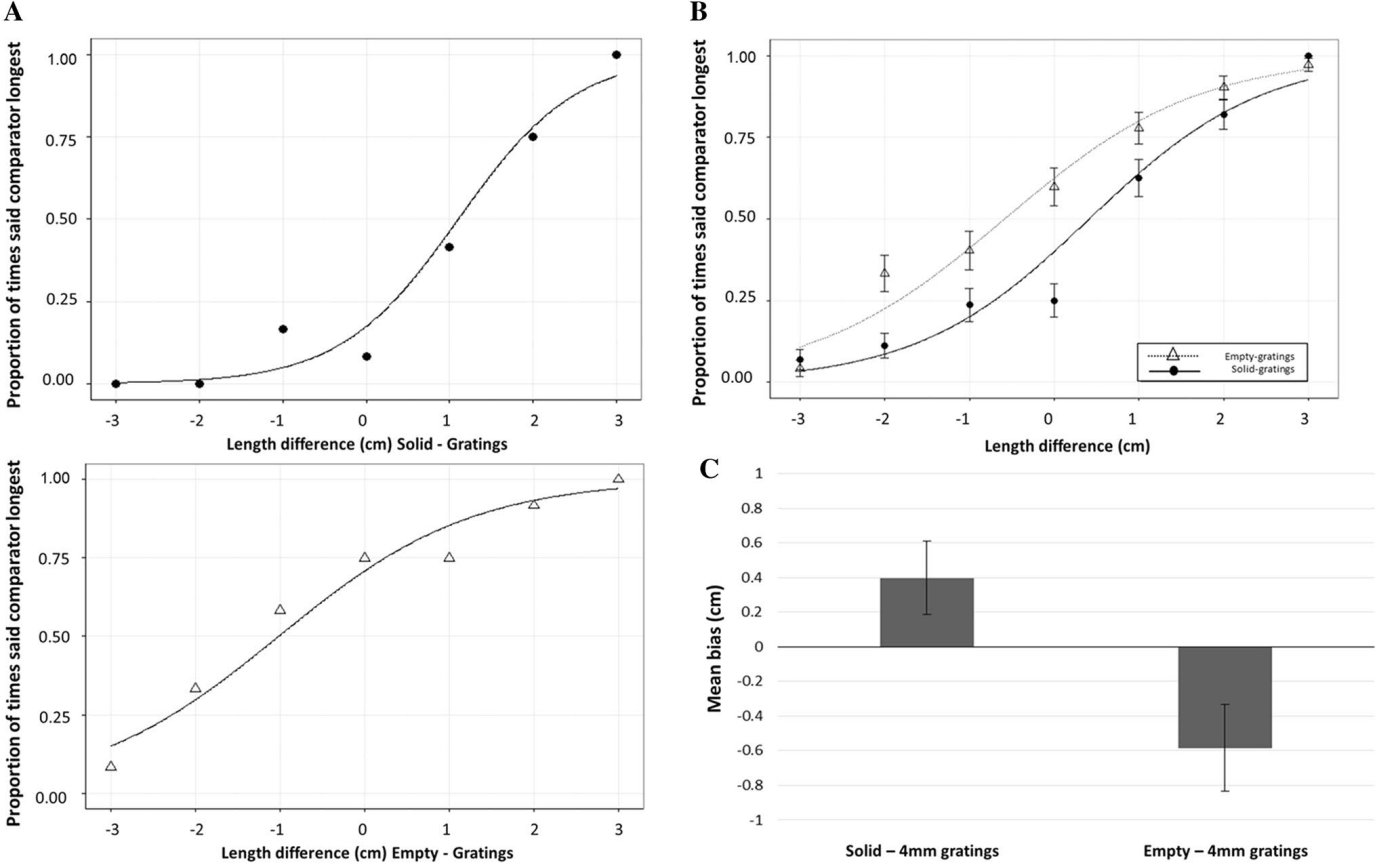
Results of Experiment 1: **a** Cumulative Gaussian curve for one participant in the solid-gratings group (*top*) and one participant in the empty-gratings group (*bottom*) **b** Mean Cumulative Gaussian curves for each group. **c** Mean bias in cm for each group. Positive biases indicate that gratings were perceived as longer than the comparator (standard filled space illusion) whereas negative biases indicate that the comparator was perceived as longer than the gratings (reverse filled space illusion). All *error bars* show ± one standard error of the mean

The proportion of times that participants stated that the comparator was longer than the gratings was plotted against the length differences. Cumulative Gaussians were then fitted for each participant, from which biases and 75 % discrimination thresholds were calculated. Examples from the solid-gratings and empty-gratings groups are shown in Fig. 3a. Mean Cumulative Gaussians for each group are shown in Fig. 3b. Positive biases indicate that the empty or solid comparator had to be longer than the grating to feel equivalent in length, i.e. the standard filled space illusion for touch. Negative biases indicate that the grating had to be longer than the comparator to feel equivalent in length, i.e. the reverse illusion reported by Parrish (1893).

Mean biases are shown in Fig. 3c. One-sample *t* tests showed that biases were not significantly greater than zero in the solid-gratings group (*m* = 0.4 cm, SD = 0.5 cm), *t*(5) = 1.9, *p* = 0.12, and they were not significantly lower than zero in the empty-gratings group (*m* = −0.6 cm, SD = 0.6 cm), *t*(5) = −2.3, *p* = 0.066, although this was a marginal result. However, an independent samples *t* test revealed that biases for the empty-gratings group were significantly less than biases in the solid-gratings group, *t*(10) = − 3.0, *p* = 0.013. Mean 75 % discrimination thresholds were 1.5 cm (SD = 0.3 cm) for the solid-gratings group and 0.6 cm (SD = 0.6 cm) for the empty-solid group.

The biases for the solid-gratings and empty-gratings groups differed significantly from each other. For the solid-gratings group, solid surfaces were, on average, 0.4 cm longer than 4-mm gratings when they were perceived as equal in length, whilst for the empty-gratings group, empty stimuli were, on average, 0.6 cm shorter than 4-mm gratings when they were perceived as equal in length. Our results show that solid and empty comparators have different influences on the perceived length of gratings, so the nature of the comparator matters in this task. Since our participants actively explored all of the stimuli in the same way, our results do not support Parrish’s (1893) assertion that a lack of movement caused the reversal of the filled space illusion in touch that she reported. Instead, our results suggest that the standard illusion weakens, and may reverse, when cutaneous information is minimised for empty space comparators.

## Experiment 2

In Experiment 2 we replicated Experiment 1 by comparing length perception when gratings were compared to a solid surface or empty space but using a stronger within-subjects design. In addition we included a second set of gratings where the bars were 8 mm apart, thus reducing the cutaneous information relative to the 4-mm gratings. Based on the results of Experiment 1 and of previous studies of the filled space illusion in touch (Parrish 1893; Sanders and Kappers 2009), we expected that gratings would be perceived as longer than smooth, solid surfaces, but those gratings would be perceived as shorter than empty space. We also expected that the standard filled space illusion would be stronger for the denser, 4-mm gratings than for the 8-mm gratings, as reported by Dresslar (1893) and Sanders and Kappers (2009).

## Method

### Participants

Eight new participants (mean age = 25 years; 5 males) either volunteered or were given course credit for their time.

### Apparatus and stimuli

The apparatus and stimuli were identical to those used in Experiment 1 except that the set of 8-mm gratings stimuli was also used.

### Design and procedure

The procedure was the same as that in Experiment 1 except as specified here. Instead of a between-subjects design, we used a within-subjects design. Participants completed four conditions: empty-4-mm gratings, empty-8-mm gratings, solid-4-mm gratings and solid-8-mm gratings. There were 84 trials per condition, so each participant completed 336 trials across four sessions of 84 trials. Trials were presented in a different random order in each session for each participant and each block was made up of a mix of trials from all four conditions. Within each session, participants were offered a short break after 42 trials. All sessions were completed within 3 weeks and each session lasted approximately 1 h.

### Results and discussion

**Fig. 4:**
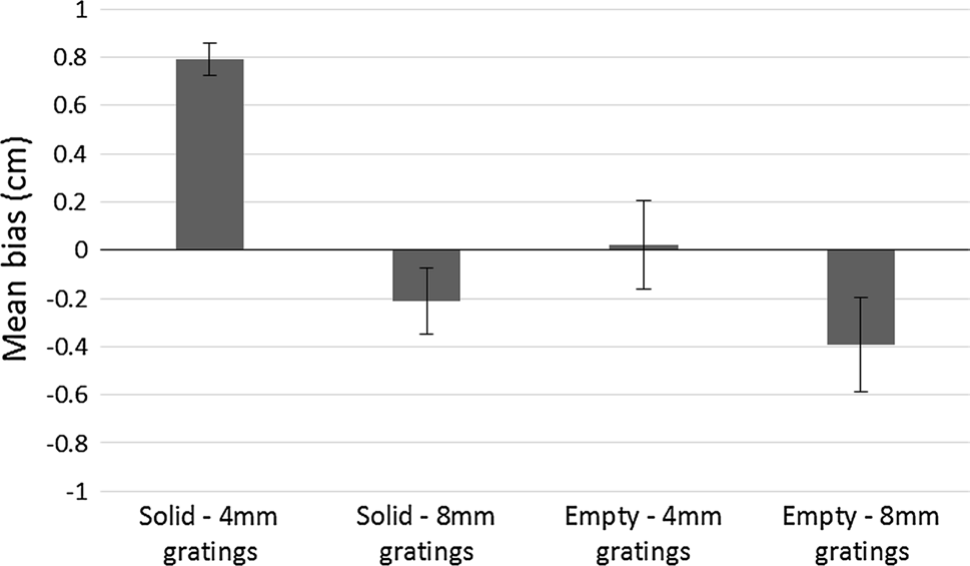
Mean biases in each condition in Experiment 2. Positive biases indicate show that gratings were perceived as longer than the comparator (standard filled space illusion), whereas negative biases indicate that the comparator was perceived as longer than the gratings (reverse filled space illusion). *Error bars* represent ± standard errors of the mean

Biases and 75 % discrimination thresholds were calculated as in Experiment 1. Mean biases are shown in Fig. 4. We conducted a repeated measures ANOVA on the biases with comparator (empty/solid) and spatial period (4/8 mm) as factors. Biases for solid comparators (*m* = 0.3 cm, SD = 0.6 cm) were not greater than for empty comparators (*m* = −0.2 cm, SD = 0.6 cm), *F*(1,7) = 4.6, *p* = 0.070, η_p_^2^ = 0.4, although this was a marginal result. Biases for 4-mm gratings (*m* = 0.4 cm, SD = 0.5 cm) were significantly greater than for 8-mm gratings (*m* = −0.3 cm, SD = 0.5 cm), *F*(1,7) = 52.5, *p* < 0.001, η_p_^2^ = 0.9. There was also a significant comparator × spatial period interaction, *F*(1,7) = 6.1, *p* = 0.043, η_p_^2^ = 0.5. To understand this interaction, post hoc paired samples *t* tests were conducted. Biases for solid-4-mm gratings (*m* = 0.8 cm, SD = 0.2 cm) were significantly greater than for solid-8-mm gratings (*m* = −0.2 cm, SD = 0.4 cm), *t*(7) = 4.2, *p* = 0.001, so increasing the spatial period of the gratings increased the strength of the standard filled space illusion. Biases for empty-4-mm gratings (*m* = 0.02 cm, SD = 0.5 cm) were significantly greater than biases for empty-8-mm gratings (*m* = −0.4 cm, SD = 0.6 cm), *t*(7) = 2.6, *p* = 0.031. Thus, empty space seemed longer when compared to 8-mm gratings than when compared to 4-mm gratings. Replicating Experiment 1, biases for solid-4-mm gratings were significantly greater than biases for empty-4-mm gratings, *t*(7) = 5.8, *p* = 0.004. However, there was no significant difference between biases for solid-8-mm gratings and empty-8-mm gratings, *t*(7) = 0.59, *p* = 0.5.

One-sample *t* tests showed that biases for solid-4-mm gratings were significantly greater than zero, *t*(7) = 11.7, *p* < 0.001. Biases for solid-8-mm gratings, *t*(7) = −1.5, *p* = 0.17, empty-4-mm gratings, *t*(7) = 0.12, *p* = 0.9 and empty-8-mm gratings, *t*(7) = −2.0, *p* = 0.08, were not significantly different from zero. Mean 75 % discrimination thresholds were 1.8 cm (SD = 0.6 cm) for the solid-4-mm gratings condition, 0.8 cm (SD = 0.4 cm) for the solid-8-mm gratings condition, 1.2 cm (SD = 0.5 cm) for the empty-4-mm gratings condition and 0.7 cm (SD = 0.5 cm) for the empty-8-mm gratings condition.

As in Experiment 1, the results of Experiment 2 suggest that changing the comparator could influence the filled space illusion in touch as once again the biases for the solid-4-mm gratings and the empty-4-mm gratings groups differed significantly from each other. We will consider these results in more detail in “General Discussion”.

## Experiment 3

**Fig. 5:**
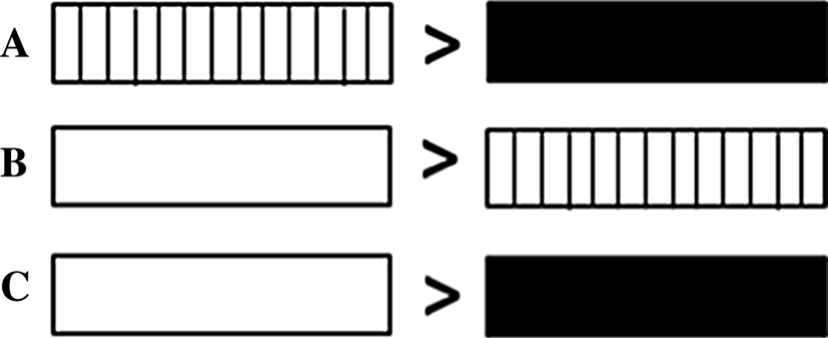
Reasoning behind Experiment 3. Though the results did not reach statistical significance, the results of Experiments 1 and 2 showed a trend where **a** gratings were perceived as longer than solid surfaces (standard filled space illusion), and **b** empty space was perceived as longer than gratings (reverse filled space illusion). Thus, in Experiment 3 we predicted that **c** empty space would be perceived as longer than solid surfaces

Experiments 1 and 2 varied the nature of the comparator when the length of gratings was estimated. In Experiment 3, we instead directly compared how long empty spaces felt relative to smooth, solid surfaces. Participants actively explored all stimuli in Experiments 1 and 2 and they used the same movements to feel gratings, solid extents and empty extents. Thus, we assume that the reversal of the filled space illusion when comparing gratings to empty space reported by Parrish (1893) was not due to changes in kinaesthetic inputs. If the effects reported in Experiments 1 and 2 occurred because length is overestimated when cutaneous information is minimal for empty extents, then the length of empty extents should continue to be overestimated when they are compared directly to solid extents, as illustrated in Fig. 5. Thus, in Experiment 3^1^ we tested the prediction that gratings are not required to elicit a bias in length estimation by active touch.

## Method

### Participants

We tested six of the eight participants who took part in Experiment 2 (the Expert group, mean age = 26 years; 4 males) and six new, naïve participants (the Naïve group, mean age = 27 years; 1 male). Participants either volunteered or were given course credit for their time.

### Apparatus and stimuli

The apparatus and stimuli were identical to those used in Experiment 1 and 2, except that only the empty and solid stimuli were used.

### Procedure

The procedure was similar to that used in Experiments 1 and 2 except that on each trial both an empty and a solid stimulus were presented and no gratings stimuli were used. On half of the trials the 12 cm reference length was an empty extent and on half of the trials it was a solid extent. Length differences were calculated as [solid–empty]. For example, the length difference of −2 cm was specified by the following four combinations: solid 12 cm then empty 14 cm, solid 10 cm then empty 12 cm, empty 14 cm then solid 12 cm and, finally, empty 12 cm then solid 10 cm. There were 84 trials in total and the experiment took approximately 1 h.

### Results and discussion

**Fig. 6:**
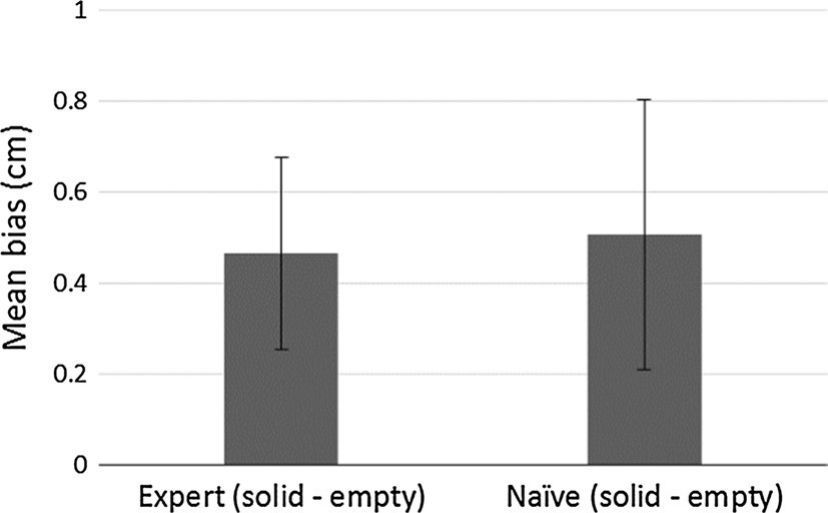
Mean biases in each condition in Experiment 3. Positive biases indicate that empty space was perceived as longer than solid surfaces. *Error bars* represent ± standard errors of the mean

Biases and 75 % discrimination thresholds were calculated in the same way as in Experiments 1 and 2. Positive biases indicate that solid surfaces had to be longer than empty space to feel equivalent in length. Mean biases are shown in Fig. 6. An independent samples *t* test showed that biases for the Expert (*m* = 0.5 cm, SD = 0.5 cm) and Naïve (*m* = 0.5 cm, SD = 0.7 cm) groups did not differ from each other, *t*(10) = −0.12, *p* = 0.8. One-sample *t* tests showed that biases were significantly greater than zero in both the Expert group, *t*(5) = 2.2, *p* < 0.001, and the Naïve group *t*(5) = 1.7, *p* < 0.001. Mean 75 % discrimination thresholds were 1.4 cm (SD = 0.7 cm) for the Expert group and 1.8 cm (SD = 0.8 cm) for the Naïve group. In support of the indirect evidence from Experiments 1 and 2, the results of Experiment 3 provided direct evidence that extents in empty space are perceived as longer than extents of solid surfaces.

## Experiment 4

Experiments 1 and 2 suggested that haptic length estimation is influenced by whether gratings are compared to smooth, solid surfaces or empty space. Experiment 3 showed that empty space is overestimated relative to smooth, solid surfaces. Together these results show that, for touch, it matters whether the lengths to be estimated are gratings or solid surfaces or empty space. In Experiment 4, we tested whether stimulus type also influenced length estimation in vision. Both vision and touch are efficient at processing spatial information (Lawson 2009) and the filled space illusion has been studied extensively in vision (e.g. Bulatov and Bertulis 1999; Coren and Girgus 1978; Deregowski and McGeorge 2006; Eriksson 1970; Mikellidou and Thompson 2011, 2014). However, as for touch, the distinction between comparators consisting of smooth, solid surfaces versus empty space does not usually appear to have been carefully considered. Testing whether the nature of the comparator matters in vision as well as touch may help to establish why the comparator seems to matter for touch. Specifically, if the nature of the comparator does not matter for vision, then the effects reported in Experiments 1–3 were likely the result of modality-specific processes in touch. In Experiment 4, we used stereoscopy to present stimuli which were empty extents, gratings or smooth, solid surfaces.

## Method

### Participants

Sixteen participants (mean age = 21 years; 6 males) took part for course credit. Participants either volunteered or were given course credit for their time.

### Stimuli and apparatus

In Experiment 4, the stimuli lengths covered a narrower range (10.5–13.5 cm in 0.5 cm increments rather than 9–15 cm in 1 cm increments as used in Experiments 1–3) since visual length estimation was expected to be better than haptic length estimation. Stimuli were extents generated in PsychoPy (Peirce 2007, 2008, 2015) and were presented on an LCD 3D screen (51 cm × 29 cm, resolution = 1920 × 1080 pixels). The extents were filled with a solid yellow block for the solid stimuli, with yellow bars for the gratings stimuli and with a single yellow bar at each end of the extent for the empty space stimuli. All bars were 1 mm wide and all stimuli were 30 mm tall on the screen. For the gratings, the bars were added at 4-mm or 8-mm intervals and, unlike Experiments 1–3, the start and end bars were the same width as the grating bars. As a result, the lengths of the gratings were generally shorter than that specified because bars were only added up to the specified length. For example, when the length of 11 cm was specified, both the solid and empty stimuli were exactly 11 cm on the screen but the 4-mm gratings were 10.8 cm long and the 8-mm gratings were 10.4 cm long. An exception was the reference length where all the stimuli were exactly 12 cm long. This meant that for the solid-4-mm gratings and empty-4-mm gratings conditions there were 13 length differences (−1.5, −1.2, −1, −0.8, −0.5, −0.4, 0, 0.5, 0.8, 1, 1.2, 1.5 and 1.6 cm) and for the solid-8-mm gratings and empty-8-mm gratings conditions there were 10 length differences (−1.5, −1, −0.8, −0.5, 0, 0.5, 0.8, 1, 1.5 and 1.6 cm). Comparisons between the specified lengths and the actual lengths on the screen can be found in “Appendix”.

**Fig. 7:**
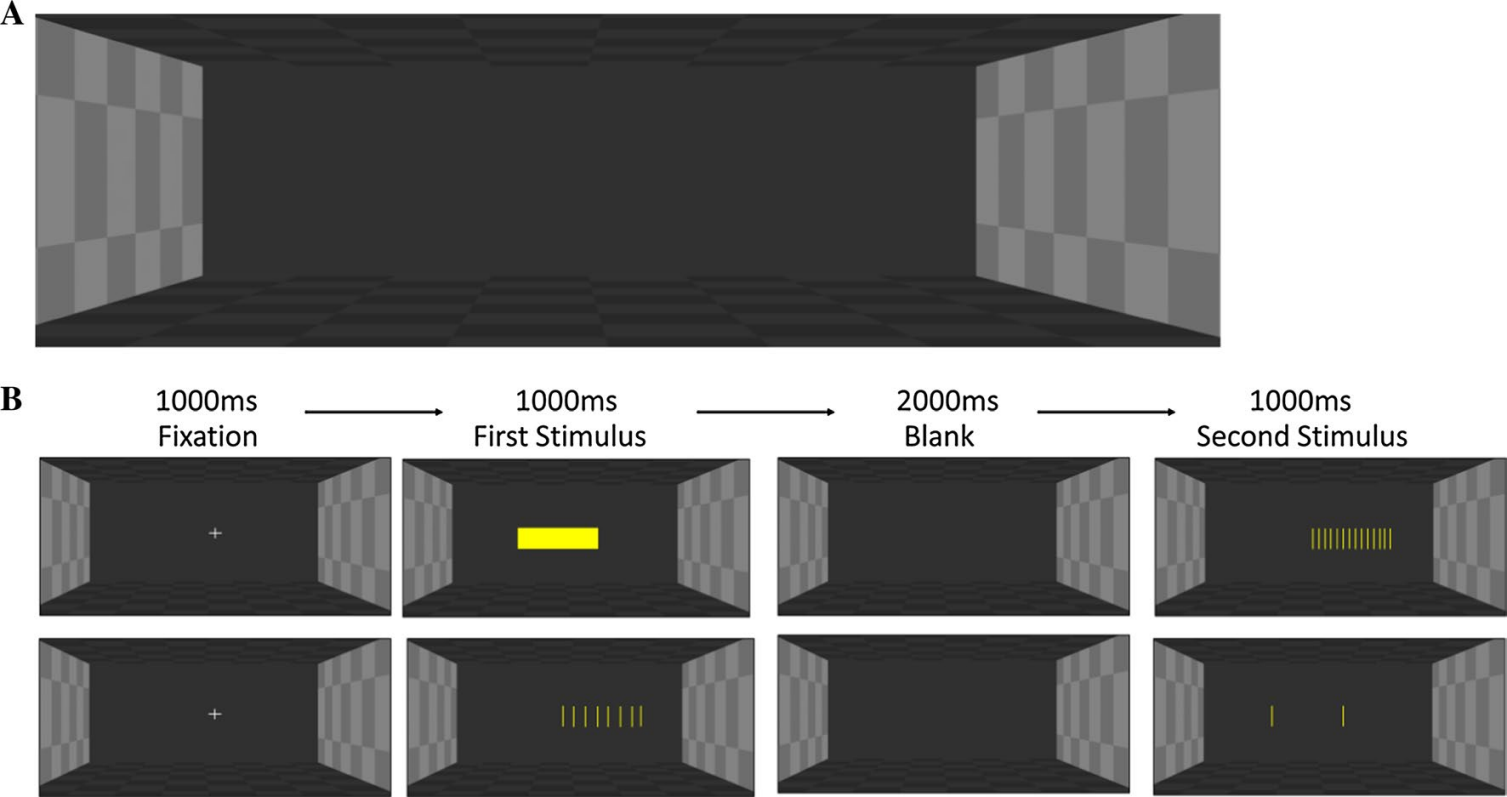
**a** View of the box in which stimuli were presented in Experiment 4. **b** An illustration of the time course of two example trials in Experiment 4 using the 12 cm extents: *top* a solid then 4-mm gratings trial; *bottom* an 8-mm gratings then empty trial

Participants sat approximately 60 cm from the screen. To present the stereoscopic scene, two images were displayed superimposed on the screen with polarised filters. The images were left-eye and right-eye versions on an image of the inside of an empty box, as shown in Fig. 7a. Throughout the experiment, participants wore 3D passive glasses, which also contained polarised filters. Since light only passed through the filter which was similarly polarised, only one of the images was presented to each eye, achieving the stereo effect. A stereo disparity of 20 pixels (50 mm) was added between the left-eye and right-eye images. The sides of the box had a light grey chequer pattern, and the top and bottom had a dark grey pattern. The back was solid grey to ensure that participants could not use any pattern information to estimate length. The stimuli were presented on the screen plane (with zero disparity) as shown in Fig. 7b, and were perceived to be floating inside the box. During experimental trials, the first stimulus was laterally shifted by 2 cm to the right of the centre of the screen and the second stimulus by 2 cm to the left of the centre or vice versa.

### Design and procedure

The design replicated that of Experiment 2 so participants compared the length of gratings with either a 4-mm or 8-mm spatial period to comparators which were either solid surfaces or empty extents. We used a two-alternative forced-choice design with the method of constant stimuli, as shown in Fig. 7b. A short sound signalled the beginning of each trial. Participants then saw a central fixation cross for 1000 ms followed by the first stimulus, which stayed on the screen for 1000 ms. The screen was then cleared and only the background was visible for 2000 ms. The second stimulus was then presented for 1000 ms. Participants were prompted to choose which stimulus was longer. They responded by pressing “8” on the number pad of the keyboard for the first stimulus or “2” for the second stimulus. No feedback was given.

Prior to completing the main experiment, participants completed five practice trials in each of which the 12 cm solid stimulus was followed by the 12 cm 4-mm gratings stimuli. As in Experiment 2 there were four blocks of 84 trials, giving 336 trials in total but all four blocks were completed within a single session. Blocks were made up of trials from all four conditions, trial order was fully randomised for each participant, and participants were offered a break after every block. The experiment lasted approximately 45 min.

### Results and discussion

**Fig. 8:**
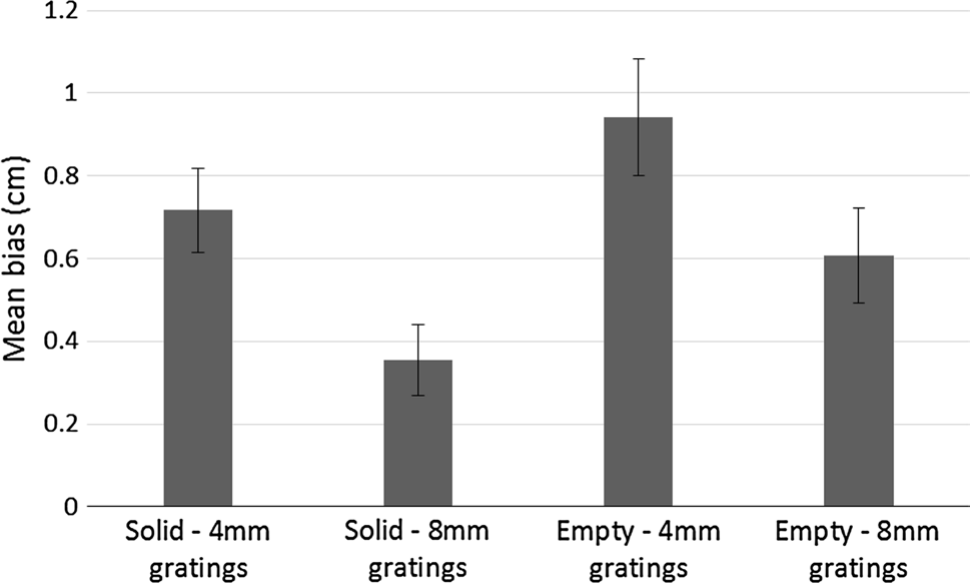
Mean biases in each condition of Experiment 4: Positive biases indicate that gratings were perceived as longer than the comparator (standard filled space illusion). *Error bars* represent ± one standard error of the mean

Biases and slopes were calculated in the same way as in Experiments 1–3. Mean biases are shown in Fig. 8. Positive biases indicate that the empty or solid comparator had to be longer than the grating to feel equivalent in length, i.e. the standard filled space illusion for vision. A repeated measures ANOVA where comparator (solid/empty) and spatial period (4/8 mm) were within-subjects factors revealed that biases for empty comparators (*m* = 0.8 cm, SD = 0.5 cm) were marginally significantly greater than biases for solid comparators (*m* = 0.5 cm, SD = 0.4 cm), *F*(1,15) = 4.2, *p* = 0.059, η_p_^2^ = 0.2. In addition, biases for 4-mm gratings (*m* = 0.8 cm, SD = 0.4 cm) were significantly greater than for 8-mm gratings (*m* = 0.5 cm, SD = 0.1 cm), *F*(1,15) = 9.4, *p* = 0.008, η_p_^2^ = 0.4. There was no significant comparator × spatial period interaction, *F*(1,15) = 0.06, *p* = 0.8, η_p_^2^ = 0.004.

One-sample *t* tests showed that mean biases were significantly greater than zero for solid-4-mm gratings (*m* = 0.7 cm, SD = 0.4 cm), solid-8-mm gratings (*m* = 0.4 cm, SD = 0.3 cm), empty-4-mm gratings (*m* = 0.9 cm, SD = 0.6 cm) and empty-8-mm gratings (*m* = 0.6 cm, SD = 0.5 cm), *t*(15) = 7.1, *t*(15) = 4.1, *t*(15) = 6.7 and *t*(15) = 5.3, respectively, all *p* < 0.001. Mean 75 % discrimination thresholds were 1.4 cm (SD = 0.3 cm) for the solid-4-mm gratings condition, 1.2 cm (SD = 0.3 cm) for the solid-8-mm gratings condition, 1.2 cm (SD = 0.8 cm) for the empty-4-mm gratings condition and 1.3 cm (SD = 0.4 cm) for the empty-8-mm gratings condition.

The results of Experiments 1–3 for touch showed that empty space felt longer than solid extents of the same length, and there was also a trend for empty space to feel longer than gratings. In contrast, in Experiment 4 for vision the standard filled space illusion was found whether gratings were compared to empty or solid extents, with the strength of the filled space illusion marginally greater when the comparator was empty rather than solid.^2^ This suggests that the nature of the comparator matters only for the filled space illusion in touch.

## General discussion

Previous researchers have usually, without comment, tested the filled space illusion in touch by asking participants to estimate the length of extents filled with gratings relative to smooth, solid comparators (e.g. Sanders and Kappers 2009: Suzuki and Arashida 1992) rather than relative to truly empty comparators. However, our results indicate that the nature of the comparator matters when people use touch to compare the length of extents. However, for vision we found that gratings were estimated as longer whether they were compared to empty or to solid extents. Only for touch did empty space seem longer than other extents. We suggest that this effect can be explained in terms of the dual nature of haptic touch, as explained below.

To our knowledge, Parrish (1893) was the first to report that the filled space illusion can be reversed in touch. Her participants kept their arms stationary throughout her experiment so Parrish concluded that the reverse filled space illusion arose when arm movements were restricted. However, both kinaesthetic and cutaneous inputs were restricted in her experiment so an alternative reason for the reversal could have been that cutaneous, not kinaesthetic, cues were restricted. In Experiments 1 and 2 reported here, kinaesthetic inputs were constant because participants made the same hand and arm movements on every trial. Only cutaneous information varied across the different stimuli. Our results suggest that the reverse filled space illusion reported by Parrish may not have arisen from a lack of movement as she argued. Instead, we propose that the minimal cutaneous input available from empty extents made them feel longer than both gratings and solid extents, as elaborated below.

Dupin et al. (2015) investigated how cutaneous and kinaesthetic cues are integrated in touch by providing only cutaneous information to one hand whilst providing only kinaesthetic information to the other hand. They found that participants were able to integrate the separated cutaneous and kinaesthetic signals into a coherent representation in order to identify the size of virtual triangles. When cutaneous and kinaesthetic cues were presented to different hands, participants seemed to rely more on movement/presentation duration relative to when the two cues were presented to the same hand. Dupin et al. suggested that the strategy used to judge size weighted cutaneous and kinaesthetic cues differently depending on how the information was presented.

**Fig. 9:**
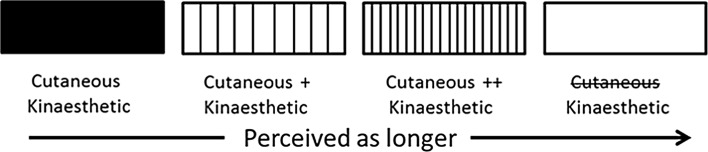
Diagram summarising our interpretation of the results of Experiments 1–3. The increased cutaneous input from the low density gratings means that they are perceived as longer than solid surfaces. Feeling high density gratings provides even more cutaneous input so these are perceived as still longer. However, when cutaneous input is minimal, for the empty extents, participants may switch strategies for length estimation and rely only on kinaesthetic information. This may lead to empty space being perceived as longer than the other kinds of stimuli

Dupin et al’s conclusions are relevant for interpreting the results of Experiments 1–3 here. Our participants may also have used different strategies to estimate length depending on the availability of kinaesthetic and cutaneous cues. The weighting of kinaesthetic and cutaneous information matters because spatial information acquired kinaesthetically and cutaneously may not be processed in the same way and so length estimates may differ depending on the extent to which each cue is used. Increased cutaneous input may increase perceived length so smooth, solid surfaces feel shorter than 8-mm gratings which, in turn, feel shorter than 4-mm gratings, as has already been reported by Sanders and Kappers (2009) and as illustrated in Fig. 9. Previous work has also suggested that estimates of length and distance are overestimated when observers rely on kinaesthetic information alone (Ebied et al. 2003; Jastrow 1886). Thus, for empty stimuli participants may rely primarily on kinaesthetic cues to estimate length, which may lead to these stimuli being perceived as longer than all other stimuli (Ebied et al. 2003; Jastrow 1886).

Why might extents feel longer when we are primarily reliant on kinaesthetic, rather than cutaneous, information? One possibility is that the extra effort involved in keeping the finger raised and moving smoothly through empty space may explain why empty space was perceived as longer than solid surfaces.^3^ Other haptic illusions, for example the radial–tangential effect (RTE; Davidon and Cheng 1964; Wong 1977), have been suggested to reflect differences in the amount of effort required to execute particular arm movements (McFarland and Soechting 2007). The RTE refers to the bias to overestimate distances moved when making arm movements of equal distance radially (away from or towards the body) compared to tangentially (across the front or side of the body, keeping distance from the body constant). For example, Debats et al. (2010) used a simulated arm to test whether differences in the moment arm (the horizontal distance from the shoulder joint to the arm’s centre of mass) affected perceived length for radial and tangential movements. The moment arm determines the amount of effort required to counteract gravitational forces and keep the arm at a constant vertical height. The moment arm changes during radial, but not tangential, movements, and more effort is required to counteract gravitational forces acting on the arm during radial movements, leading to an overestimation of length (Debats et al. 2010). A similar mechanism may explain why empty space was perceived as longer than solid surfaces in the present experiments. The finding that the filled space illusion did not reverse in vision (Experiment 4) supports this modality-specific account.

Another effect which may be related to our findings was reported by Bergmann Tiest and Hayward (2015). They found that when observers compared the size of solid circular discs (exploring an object from the outside) and holes (exploring an object from the inside) of equal size using their index finger, there was a non-significant tendency for them to perceive the holes as larger than the discs. However, we believe that it is unlikely that our results are due only to this effect. First, our participants did not only explore the inside of the empty stimuli in the empty space conditions of Experiments 1–3. Instead they initially felt the outside of the solid end bars of the stimuli as they moved their finger from the start to the endpoint and vice versa. Second, our participants were instructed to consider the full length of the stimuli, and this included the end bars of the empty stimuli.

Our results did not fully replicate Sanders and Kappers (2009) because the positive bias found when comparing smooth, solid surfaces to gratings was not significantly different from zero in Experiment 1 or, for 8-mm gratings, in Experiment 2. This suggests that the standard filled space illusion in touch may not be as reliable and strong as the literature has previously suggested. In addition, the negative bias for comparing empty space to gratings was not significantly different from zero in Experiment 1 or 2. We suggest that future research investigating the relative importance of kinaesthetic and cutaneous cues to haptic length perception would be better served by a more sensitive and reliable task. Nevertheless, we did consistently observe differences when we compared haptic estimations involving smooth, solid surfaces to those involving empty space in Experiments 1, 2 and 3 here.

In the present work, we found that gratings are perceived as longer when compared to solid surfaces than to empty space. The pattern of our data was consistent with Parrish (1893), suggesting that the filled space illusion might reverse when gratings are compared to empty space rather than to smooth, solid surfaces (Experiments 1 and 2). Supporting this hypothesis, we found that empty space appears longer than smooth, solid surfaces (Experiment 3). We did not find a comparable effect of the nature of the comparator for vision (Experiment 4), suggesting that the difference between empty space and solid surfaces in touch arises from modality-specific effects on how length is processed. We propose that the nature of the comparator may matter in touch due to the relative availability and use of cutaneous and kinaesthetic cues, and perhaps also the greater effort required to keep the finger aloft whilst exploring empty space. In conclusion, our sense of touch perceives the length of empty space, solid surfaces and gratings differently.

## Appendix

**Table:**

Specified length (cm)	Solid blocks (cm, on screen)	Empty space (cm, on screen)	4-mm gratings (cm, on screen)	8-mm gratings (cm, on screen)
10.5	10.5	10.5	10.4	10.4
11	11	11	10.8	10.4
11.5	11.5	11.5	11.2	11.2
12	12	12	12	12
12.5	12.5	12.5	12.4	12
13	13	13	12.8	12.8
13.5	13.5	13.5	13.2	12.8

The actual lengths presented in Experiment 4 for the four type of stimuli used

## Footnotes

1 There was a second condition in Experiment 3 which all participants completed. Here participants gained extra cutaneous information by moving their finger along the smooth, solid base of the apparatus between the startpoint and the start of the stimulus and also between the end of the stimulus and the endpoint rather than moving their finger above the base through air. Their finger thus only passed through empty space when moving between the start and end bars of the empty extents. These results are not reported here because some participants reported using a different strategy in this condition making it difficult to interpret in a theoretically meaningful manner.

2 This difference could have resulted from the irradiation illusion in which a bright extent appears longer than a darker extent of equal length (Coren and Girgus 1978; Helmholtz 1925; Long and Murtagh 1984). The irradiation illusion would make the solid comparator appear longer than the empty comparator, and this effect would work to weaken the filled space illusion for solid comparators relative to empty comparators.

3 We thank an anonymous reviewer for this suggestion.
